# Resolving molecular diffusion and aggregation of antibody proteins with megahertz X-ray free-electron laser pulses

**DOI:** 10.1038/s41467-022-33154-7

**Published:** 2022-09-21

**Authors:** Mario Reiser, Anita Girelli, Anastasia Ragulskaya, Sudipta Das, Sharon Berkowicz, Maddalena Bin, Marjorie Ladd-Parada, Mariia Filianina, Hanna-Friederike Poggemann, Nafisa Begam, Mohammad Sayed Akhundzadeh, Sonja Timmermann, Lisa Randolph, Yuriy Chushkin, Tilo Seydel, Ulrike Boesenberg, Jörg Hallmann, Johannes Möller, Angel Rodriguez-Fernandez, Robert Rosca, Robert Schaffer, Markus Scholz, Roman Shayduk, Alexey Zozulya, Anders Madsen, Frank Schreiber, Fajun Zhang, Fivos Perakis, Christian Gutt

**Affiliations:** 1 Department of Physics, AlbaNova University Center, Stockholm University, SE-106 91 Stockholm, Sweden; 2 Institut für Angewandte Physik, Universität Tübingen, Auf der Morgenstelle 10, 72076 Tübingen, Germany; 3 Department Physik, Universität Siegen, Walter-Flex-Strasse 3, 57072 Siegen, Germany; 4 ESRF - The European Synchrotron, 71 Avenue des Martyrs, CS 40220, 38043 Grenoble Cedex 9, France; 5 Institut Laue-Langevin, 71 Avenue des Martyrs, CS 20156, 38042 Grenoble Cedex 9, France; 6 European X-Ray Free-Electron Laser Facility, Holzkoppel 4, 22869 Schenefeld, Germany

**Keywords:** Nanoscale biophysics, Free-electron lasers, Imaging techniques, Proteins

## Abstract

X-ray free-electron lasers (XFELs) with megahertz repetition rate can provide novel insights into structural dynamics of biological macromolecule solutions. However, very high dose rates can lead to beam-induced dynamics and structural changes due to radiation damage. Here, we probe the dynamics of dense antibody protein (Ig-PEG) solutions using megahertz X-ray photon correlation spectroscopy (MHz-XPCS) at the European XFEL. By varying the total dose and dose rate, we identify a regime for measuring the motion of proteins in their first coordination shell, quantify XFEL-induced effects such as driven motion, and map out the extent of agglomeration dynamics. The results indicate that for average dose rates below 1.06 kGy μs^−1^ in a time window up to 10 μs, it is possible to capture the protein dynamics before the onset of beam induced aggregation. We refer to this approach as *correlation before aggregation* and demonstrate that MHz-XPCS bridges an important spatio-temporal gap in measurement techniques for biological samples.

## Abstract (web summary)

The European X-ray FreeElectron Laser Facility generates ultrashort hard X-ray pulses with megahertz repetition rate. Here, the authors probe the dynamics of dense antibody protein (Ig-PEG) solutions using megahertz X-ray photon correlation spectroscopy at the European XFEL.

## Introduction

**Fig. 1:**
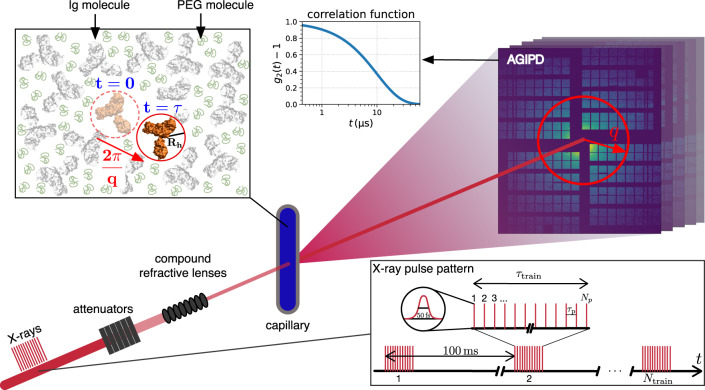
Scheme of the experiment. Highly concentrated solutions of immunoglobulin (Ig) with polyethylene glycol (PEG) are measured in quartz capillaries. An individual Ig molecule has a hydrodynamic radius of *R*_*h*_ = 5.5 nm. Megahertz X-ray photon correlation spectroscopy (MHz-XPCS) measurements are performed by using trains of X-ray pulses, which illuminate the sample. The spacing between two pulses within a train is *τ*_*p*_ and was varied between 443 and 886 ns where a train contains *N*_*p*_ individual X-ray pulses. The length of an individual X-ray pulse is ≤50 fs^50^. A new train is delivered every 100 ms. The train duration is determined by the number of pulses per train and the delay time between the pulses : *τ*_train_ = (*N*_*p*_ − 1)*τ*_*p*_. The longest train duration during the experiment was *τ*_train,max_ = (144 − 1) × 886 ns ≈ 127 μs. For a period of 100 ms − *τ*_train_, the sample is not illuminated by X-rays. By analyzing sequential X-ray scattering patterns measured with the adaptive gain integrated pixel detector (AGIPD), information about the dynamics of the sample can be obtained in the form of intensity auto-correlation functions calculated from fluctuating speckle patterns. A measurement consists of a series of *N*_train_ individual trains (see Table 1).

The European X-ray Free-Electron Laser Facility (EuXFEL) is the first X-ray free electron laser (XFEL) generating ultrashort hard X-ray pulses with megahertz repetition rate. Megahertz X-ray photon correlation spectroscopy (MHz-XPCS)^1–3^ makes use of this high repetition rate and the high degree of transverse coherence to measure diffusive dynamics with (sub-) microsecond temporal resolution. In biological systems, typical diffusion coefficients in dense cellular environments range from *D*_0_ ≈ 0.1 to 10 nm^2^ μs^−1^^4–9^ which requires to resolve time scales from *τ* ≈ 0.5 to 5 μs (see Fig. 1) in order to trace the complex many-body interactions between proteins and the solvent on molecular length scales. This window of length and time scales is not accessible by optical techniques such as dynamic light scattering, which measures longer length scales (micrometers), or neutron spectroscopy techniques such as neutron spin echo or inelastic neutron scattering, which typically measure on faster time scales of nanoseconds and below. Clearly, experimental techniques are needed to close this gap and measure collective dynamics on microsecond time scales and nanometer length scales. By analyzing fluctuating X-ray speckle patterns, MHz-XPCS is potentially capable of closing this gap, as we demonstrate here, and enables us to gain information on equilibrium and out-of-equilibrium collective dynamics of protein solutions.

Protein dynamics in crowded environments are particularly relevant in the context of intracellular transport in the cytoplasm of eukaryotic cells^10^, phase transitions in biomolecular condensates^11–13^, aggregation phenomena^14,15^, and drug production^16^. In highly concentrated environments, the dynamics differ significantly from that of a dilute system, whereas the exact mechanisms that influence the dynamics on different time scales are not yet fully understood^6,7,17^. It was found that in vivo dynamics in cells exhibit tremendously reduced diffusion compared to in vitro measurements of diluted proteins in buffer solutions^18–25^. It is believed that the level of slowing-down depends on the particular system and possibly additional crowding agents^6,18,26–28^. In addition to excluded volume effects^29,30^, there can be contributions from the local water dynamics of the hydration layer^31^, quinary interactions of proteins with other cytoplasmic constituents^18,19,32–35^, and transient cluster formation^36–40^ that influence intracellular protein diffusion. Also, the dynamics often exhibit anomalous behavior—i.e., non-Brownian and in particular subdiffusive dynamics^26,41,42^—making it difficult to extrapolate the dynamics from the dilute regime. Clearly, new methods are needed to directly probe diffusive dynamics in crowded biological solutions on (sub-) microsecond time scales and nanometer length scales to study these phenomena.

Radiation damage constitutes a major challenge for X-ray scattering experiments with protein solutions. Radiolysis of water and the fast distribution of the free radicals formed rapidly degrade the protein molecules. Hence, a typical upper limit of tolerable absorbed doses is estimated on the order of a few kGy in these experiments with the exact value depending on the chemical composition of the system^43–46^. Protein aggregation is a signature of beam-induced damage in protein solutions visible via changes in the X-ray scattering form factor. Aggregation processes and the spread of free radicals are both driven by diffusive dynamics and act on nano- and microsecond time scales^14,47–49^. The study of such time-dependent dynamic processes in aqueous solutions of bio-molecules when illuminated with X-rays is of considerable relevance for understanding biological aspects of ionizing radiation. In addition, MHz-XFEL experiments deliver extremely high dose rates to the sample. Utilizing MHz repetition rates and high attenuation, the X-ray pulses are delivered on (sub-) microsecond time scales such that an average dose rate on the order of several kGy per microsecond can be reached. The effects of such high dose rates on structure and dynamics of protein solutions are still unknown.

Here, we report a MHz-XPCS experiment with radiation sensitive protein solutions at the Materials Imaging and Dynamics (MID) instrument^50^ at EuXFEL. We investigate the dynamics in a concentrated bovine immunoglobulin (Ig) solution where 80% of the Ig is constituted by IgG^51,52^. Immunoglobulin is an abundant antibody protein that can be found, for instance, in the blood of animals and humans. Polyethylene glycol (PEG) is added to the solution as a depletant and induces attractive protein-protein interactions that—depending on concentration and temperature—can result in liquid–liquid phase separation (LLPS)^51,52^. This combination renders the Ig-PEG system an interesting candidate for the MHz-XPCS measurements in the context of both crowding dynamics in concentrated protein solutions and the formation of biomolecular condensates.

## Results

### Measurement scheme and data collection

We employed X-ray pulses with 443 and 886 ns delays between successive pulses corresponding roughly to repetition rates of 2.26 and 1.13 MHz, respectively. The X-ray pulses were delivered in trains of up to 200 pulses with a train frequency of 10 Hz (see Fig. 1). This time structure makes it possible to conduct MHz-XPCS measurements within a single train, while the time between subsequent trains is sufficiently long to refresh the sample via translation.

The data presented here were acquired at the MID instrument in small-angle X-ray scattering (SAXS) geometry with a pink beam, i.e., using self-amplified spontaneous emission (SASE) without a monochromator, and a photon energy of 9 keV^50^. A sketch of the experimental setup is shown in Fig. 1. The Adaptive Gain Integrating Pixel Detector (AGIPD)^53^ was placed 7.46 m behind the sample with most of the sample-detector flight path being evacuated. The Ig-PEG solutions were filled into quartz capillaries with an outer diameter of 1.5 mm and a wall thickness of 20 μm. A Linkam scientific instruments stage was used to control and stabilize the sample temperature at 298 K, which is above the binodal in the single phase regime of the Ig-PEG system^51,52^. The X-ray beam was focused to a diameter of 10 μm (FWHM) using compound refractive lenses to increase the measured speckle contrast and the signal-to-noise ratio (SNR) of the XPCS measurements^54^.

Table 1 contains a summary of the measurement parameters. The intensity of the X-rays was reduced by chemically vapor deposited (CVD) diamond attenuators of various thickness and adjusted such that the samples were exposed to the lowest possible dose while keeping the scattered intensity high enough to reach a sufficient SNR. For example, with an average pulse energy of 1.2 m J and 3925 μm CVD attenuator thickness 6.5 × 10^8^ photons per X-ray pulse illuminate the sample. The incoming flux results in an average scattering signal of less than 10^−1^ photons per pixel per image. In addition to the absolute dose also the average dose rate was varied, i.e., the absorbed dose per time, measured in kGy μs^−1^. The actual dose rate value is calculated as an average over the first ten X-ray pulses and all trains of a measurement (see “Methods”).

**Table 1:** Measurement parameters

${{{{{{{{\mathcal{D}}}}}}}}}_{{{{{{{{\rm{rate}}}}}}}}}$ (kGy μs^−1^)	*f*_FEL_(MHz)	*τ*_*p*_(ns)	*T*_cvd_(%)	〈Φ_*c*_〉(10^8^ph/pls)	*N*_train_	*N*_*p*_
1.06	1.13	886	0.6	1.59	2800	144
2.04	2.26	443	0.6	1.53	9200	200
2.55	1.13	886	1.4	3.82	2000	144
4.75	2.26	443	1.4	3.56	5200	200

${{{{{{{{\mathcal{D}}}}}}}}}_{{{{{{{{\rm{rate}}}}}}}}}$ is the average dose rate, *f*_FEL_ is the XFEL frequency, and *τ*_*p*_ is the time between successive pulses which defines the the minimum XPCS delay time. *T*_cvd_ is the transmission of the diamond attenuators which results in the average number of incident photons per X-ray pulse (ph/pls) on the sample, 〈Φ_c_〉. *N*_train_ is the number of pulse trains averaged in the analysis. *N*_*p*_ is the maximum number of pulses per train.

### Megahertz small-angle X-ray scattering (MHz-SAXS)

**Fig. 2:**
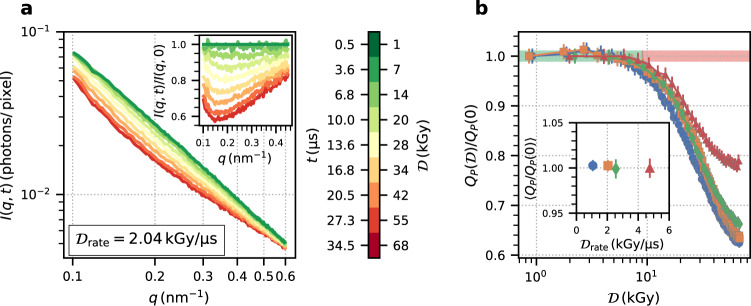
Static scattering signal of Ig-PEG. **a** The azimuthally integrated intensity, *I*(*q*, *t*), as a function of momentum transfer, *q*. The color indicates the absorbed dose ${{{{{{{\mathcal{D}}}}}}}}$ and the corresponding timescales. The data shown are acquired with a dose rate of 2.04 kGy μs^−1^. The inset displays *I*(*q*, *t*) normalized to the first pulse *I*(*q*, 0). **b** Porod invariant, ${Q}_{P}({{{{{{{\mathcal{D}}}}}}}})$, calculated from the data displayed in **a** (orange) and three additional dose rates. The data are normalized to *Q*_*P*_(0) and the error bars are calculated as the standard deviation of the normalized second pulse from unity. The inset shows the mean of ${Q}_{P}({{{{{{{\mathcal{D}}}}}}}})$ below 10 kGy for different dose rates. The error bars indicate the weighted standard deviation of *Q*_*P*_ in this range. Source data are provided as a Source Data file.

The evolution of the time-resolved SAXS signal as a function of dose and dose rate is analyzed by computing the azimuthally integrated intensity *I*(*q*, *t*) as a function of absolute momentum transfer, $q=4\pi /\lambda \sin (2\theta /2)$, where *λ* is the X-ray wavelength and 2*θ* is the scattering angle, and measurement time or dose (see Fig. 2a). The absorbed dose is proportional to the measurement time and is calculated with Eq. (5) (see “Methods”). The data displayed were recorded with a dose rate of 2.04 kGy μs^−1^, but with absolute doses varying between 1 kGy (green) and 74 kGy (red). With increasing dose, we observe significant changes in *I*(*q*, *t*), with the largest decrease of intensity visible at momentum transfers of *q* = 0.17 nm^−1^ accompanied by an increasing scattering signal at small momentum transfers. The inset shows the data normalized by *I*(*q*, 0). The Ig-PEG system exhibits a structure factor peak close to 0.62 nm^−1^ that was studied in a previous work by Da Vela et al.^51^. Additionally, the phase behavior of the Ig-PEG systems is characterized by an upper critical solution temperature of around 294 K. The overall decrease of intensity in Fig. 2 indicates that the system is moving away from the LLPS binodal in the phase diagram, presumably due to beam-induced local heating. Deeper in the single phase regime, increasingly repulsive protein-protein interactions lead to a reduced SAXS intensity. On the other hand, at *q*-values below 0.17 nm^−1^, the visible increase of *I*(*q*, *t*)/*I*(*q*, 0) indicates the formation of X-ray induced aggregation of the proteins.

We quantify the evolution of structural changes by calculating the Porod invariant

1 $${Q}_{P}(t)=\int\nolimits_{{q}_{\min }}^{{q}_{\max }}{q}^{2}I(q,t)\,{{{{{\rm{d}}}}}}q,$$

in the accessible *q*-range (*q*_min_ = 0.1 nm^−1^, *q*_max_ = 0.6 nm^−1^) as a function of dose (see Fig. 2b). ${Q}_{P}({{{{{{{\mathcal{D}}}}}}}})$ displays an initial plateau up to a maximum dose of 10 kGy after which it starts to decrease more than two percent from its initial value. At doses below 10 kGy, the protein structure seems unaffected by the X-ray illumination—at least on the length scales probed here. In this low-dose regime, we also extract the dose rate dependence of the Porod invariant by averaging the ${Q}_{p}({{{{{{{\mathcal{D}}}}}}}})$ data for ${{{{{{{\mathcal{D}}}}}}}} \; < \;{{\mbox{10}}}\,\,\,{{\mbox{kGy}}}$. The results are displayed in the inset in Fig. 2b demonstrating the absence of a dose rate dependence in the SAXS signal. This is in agreement with previous work reporting that the absolute absorbed dose is the main driver for radiation damage and dose rate effects are only weak^46^.

### Megahertz X-ray photon correlation spectroscopy (MHz-XPCS)

The disordered protein solutions give rise to a speckle pattern in the far-field when illuminated by coherent radiation. The dynamics can be studied by analyzing the speckle intensity fluctuations that are related to the microscopic motion of the protein molecules. The intensity *I*_*p*_(*q*, *t*) is measured at time *t* by pixel *p* within a concentric region of interest (ROI) of constant absolute momentum transfer. We utilized an XPCS adapted acquisition scheme in which the sample is continuously moving through the X-ray beam with a velocity of 400 μm s^−1^. The sample movement is negligible during an X-ray train ensuring illumination of the same sample spot on microsecond time scales. In between two trains the sample position offset is large enough to completely renew the sample volume, and thus to avoid accumulated damage. The low intensity scattering signal requires averaging correlation functions from many trains (between 2000 and 9000 see Table 1) to increase the SNR. Approximately 80% of the acquired trains are used for the XPCS analysis while the rest are discarded after applying filters based on diagnostics such as extremely low intensity due to the SASE fluctuations.

We compare measurements with average dose rates, ${{{{{{{{\mathcal{D}}}}}}}}}_{{{{{{{{\rm{rate}}}}}}}}}$, from 1.06 to 4.75 kGy μs^−1^. The influence of dose and dose rate on the protein dynamics can be quantified with the help of two-time correlation functions (TTCs)^55,56^ which essentially represent the correlation coefficient between speckle images taken at times *t*_1_ and *t*_2_ at momentum transfer *q*:

2 $${c}_{2}(q,{t}_{1},{t}_{2})={\left\langle \frac{{\langle {I}_{p}(q,{t}_{1}){I}_{p}(q,{t}_{2})\rangle }_{p}}{{\langle {I}_{p}(q,{t}_{1})\rangle }_{p}{\langle {I}_{p}(q,{t}_{2})\rangle }_{p}}\right\rangle }_{j}-1.$$

Here, 〈… 〉_*p*_ denotes an average over all pixels with the same absolute momentum transfer, *q*, and 〈… 〉_*j*_ denotes an average over all trains where *j* = 1…*N*_train_. The data calibration and analysis workflow for MHz-XPCS with AGIPD is described in detail in Dallari et al.^3^.

**Fig. 3:**
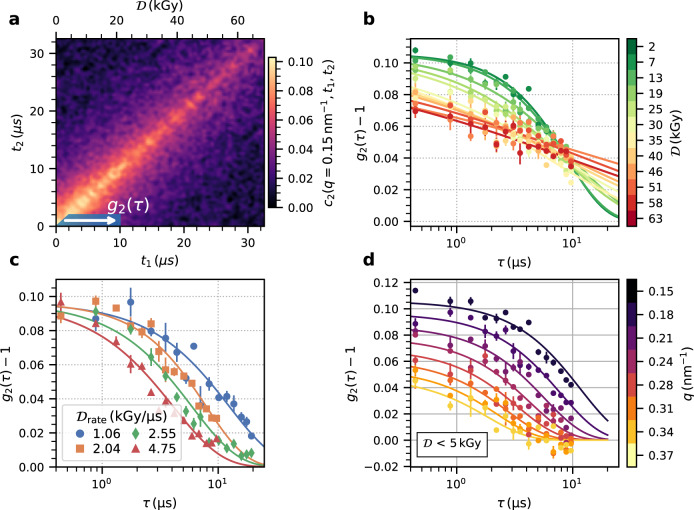
Correlation functions. **a** Two-time correlation function, *c*_2_, of Ig-PEG measured with an average dose rate of 2.04 kGy μs^−1^ for *q* = 0.15 nm^−1^. **b** Correlation functions for different initial doses (${{{{{{{{\mathcal{D}}}}}}}}}_{{{{{{{{\rm{rate}}}}}}}}}=2.04$ kGy μs^−1^, *q* = 0.15 nm^−1^). **c** Correlation functions with an initial dose below 5 kGy for different dose rates at *q* = 0.15 nm^−1^. **d** Correlation functions for different momentum transfers fitted with a *q*-squared dependent relaxation rate (${{{{{{{{\mathcal{D}}}}}}}}}_{{{{{{{{\rm{rate}}}}}}}}}=2.04$ kGy μs^−1^). The error bars represent the standard error over pixels and repetitions. Source data are provided as a Source Data file.

Figure 3a displays a TTC measured with an average dose rate of 2.04 kGy μs^−1^ at *q* = 0.15 nm^−1^. The abscissa and ordinate of the TTC show the measurement times *t*_1_ and *t*_2_, respectively, within an X-ray pulse train while the additional label at the top indicates the corresponding absorbed dose. The TTC decays with increasing distance from the diagonal describing the temporal decorrelation of the speckle fluctuations due to the sample dynamics. The fact that the diagonal does not exhibit a constant width indicates that the dynamics change throughout the measurement.

Time-resolved intra-train intensity auto-correlation functions, ${g}_{2}(q,\tau,{{{{{{{\mathcal{D}}}}}}}}({t}_{0}))$, are calculated by averaging sections of the TTCs as indicated by the white arrow in Fig. 3a:

3 $${g}_{2}(q,\tau,{{{{{{{\mathcal{D}}}}}}}}({t}_{0}))={\langle {c}_{2}(q,{t}_{1}={t}_{0}\pm {{\Delta }}t+\tau,{t}_{2}={t}_{0}\pm {{\Delta }}t)\rangle }_{{{\Delta }}t}+1.$$

To obtain the correlation function for a particular initial dose, ${{{{{{{\mathcal{D}}}}}}}}({t}_{0})$, during the measurement, the time *t*_0_ is chosen on the diagonal of the TTC in Fig. 3a, while noting that the dose increases further with each point of the correlation function. The average over Δ*t* can be seen as rebinning *c*_2_ along *t*_2_ to increase the statistics. This approach yields a set of *g*_2_(*q*, *τ*) per dose and dose rate.

The correlation functions are modeled by a Kohlrausch–Williams–Watts (KWW) function:

4 $${g}_{2}(q,\tau )=1+\beta (q)\,{{{{{\rm{e}}}}}}^{-2{({{\Gamma }}(q)\tau )}^{\alpha }},$$

where *β*(*q*) is the *q*-dependent speckle contrast^57^ (*β*(*q* = 0.15 nm^−1^) ≈ 11%) and *α* is the KWW exponent. Brownian diffusion is characterized by a quadratic *q*-dependence of the relaxation rates Γ(*q*) = *D*_0_*q*^2^, where *D*_0_ is the diffusion coefficient, and simple exponential behavior (*α* = 1). KWW exponents smaller than 1 are typically observed in supercooled liquids and gels and can indicate heterogeneous dynamics with a distribution of relaxation times^58^. A quadratic *q*-dependence and a *q*-independent KWW exponent are used to model the data. Figure 3d shows that within the experimental accuracy the KWW function describes the data well.

**Fig. 4:**
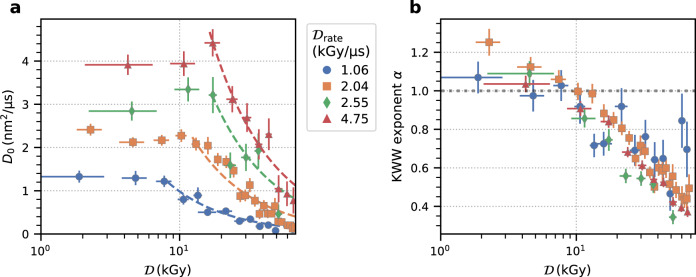
Dynamical parameters. **a** Diffusion coefficient, *D*_0_, as a function of initial dose for different dose rates indicated by the color. The dashed lines are guides to the eye. **b** KWW exponent for different dose rates as a function of total absorbed dose. Source data are provided as a Source Data file.

Figure 3c shows correlation functions for different dose rates for absolute doses below 5 kGy. The data indicates that the dynamics become faster with dose rate as the correlation functions shift to shorter time scales while the overall lineshape appears to change only slightly. This is different from the behavior observed with increasing total dose in Fig. 3b where the shape of the correlation functions drastically changes from a simple exponential decay at low doses to a highly stretched (*α* < 1) and almost logarithmic decay at higher dose values. We account for these changing KWW exponents by computing the average relaxation rate^59,60^〈Γ〉(*q*) = Γ(*q*)*α*/Γ_*f*_(1/*α*), where Γ_*f*_(*x*) is the Γ-function. Using these average relaxation rates one pair of parameters (*D*_0_, *α*) is calculated per initial dose and dose rate, where *D*_0_ = 〈Γ〉(*q*)/*q*^2^. The results are displayed in Fig. 4. The diffusion coefficients in Fig. 4a reveal a pronounced dependence on the initial dose and dose rate as already indicated by the correlation functions in Fig. 3 and are higher than expected for the base temperature of *T*_0_ = 298 K. Therefore, we denote *D*_0_ reported here is as an effective diffusion coefficient discussed in more detail in the following section. The numbers obtained for *D*_0_ are on the order of a few nm^2^ μs^−1^, which is the typical range of diffusion coefficients found for dense protein systems^6,7,26^. For a given dose rate, all diffusion coefficients follow a similar pattern as a function of initially absorbed dose: *D*_0_ is nearly independent of the initial dose up to a threshold value, above which the *D*_0_ steadily decreases. The threshold initial dose is slightly below 10 kGy for a dose rate of 1.06 kGy μs^−1^ and increases above 15 kGy for 4.75 kGy μs^−1^. The average values of *D*_0_ below these thresholds increase linearly by about a factor of four from 1.3 to 4 nm^2^ μs^−1^ with increasing dose rate. The corresponding KWW exponents do not show any pronounced dose rate dependence, but a clear dependence on the initial dose (Fig. 4b). The KWW exponents further reveal that the correlation functions exhibit a simple exponential shape (*α* = 1) for low doses, while they are increasingly stretched above 10 kGy, which approximately coincides with the dose value where a decrease in *D*_0_ becomes apparent (Fig. 4a). The simultaneous decrease of *D*_0_ and the KWW exponent for high doses points towards beam-induced aggregation of the proteins (cf. Fig. 2), which results in slower diffusion and increasingly stretched exponential behavior.

## Discussion

Our results indicate that static and dynamic properties are influenced in different ways by the intense X-ray pulses of the European XFEL. MHz-SAXS reveals that the static scattering signal—within the accessible *q*-window—is preserved below an absorbed dose of 10 kGy. This threshold value is independent of the applied dose rate (Fig. 2b inset) within the limited range of dose rates. It is noteworthy that the extremely high dose rates and microsecond time scales probed with an XFEL yield similar threshold values (≈10 kGy) as the orders of magnitude lower dose rates used at a synchrotron (≈1 kGy s^−1^^52^).

Understanding the dose rate dependence of radiation-induced effects is crucial for comparing and optimizing experiments at different radiation sources (rotating anodes, synchrotrons, XFELs). At comparably moderate dose rates of tens of Gray per second at synchrotrons, the aggregation rate of proteins was found to exhibit a dose rate dependence^46^ favoring measurements with low dose rates. On the other hand, high dose rates seem to be preferable in room temperature protein crystallography measurements^61–63^.

Generally, radiation damage in aqueous protein solutions is mainly attributed to the diffusion and successive reaction of proteins with radicals produced by radiolysis, such as OH^−^. Radiolysis itself involves a variety of different time and length scales where the radicals are not uniformly generated in the solvent, but distributed initially in nanoscale traces which broaden and diffuse into the bulk on timescales of hundreds of nanoseconds to microseconds during the chemical stage^64^. The primary yield of OH^−^ radicals is high, with 2.87 OH^−^ per 100 eV absorbed after one microsecond^65^, leading to an average of about 0.6 OH^−^ radicals per Ig protein molecule needed to induce measurable changes to the SAXS signal (see Fig. 2). The observed threshold dose of 10 kGy represents a typical time window of 9.4 μs when using a dose rate of 1.06 kGy μs^−1^. The absence of a measurable dose rate effect on this static threshold value indicates that diffusion rates of radicals, recombination, and quenching effects do not affect the overall agglomeration probability.

Additional insight can be obtained from the MHz-XPCS data, which allows to trace time-resolved non-equilibrium dynamics via the TTCs. With regard to protein diffusion the typical mean square distances probed here can be estimated via

$${g}_{2}(q,\tau )-1=\beta \exp (-{q}^{2}\langle {{\Delta }}{x}^{2}(\tau )\rangle /6)$$

yielding values of $\sqrt{\langle {{\Delta }}{x}^{2}\rangle }=\,{{\mbox{16}}}\,\,\,{{\mbox{nm}}}$ at *q* = 0.15 nm^−1^ to $\sqrt{\langle {{\Delta }}{x}^{2}\rangle }=\,{{\mbox{4}}}\,\,\,{{\mbox{nm}}}$ at *q* = 0.6 nm^−1^ at 1/*e* decay of the correlation functions. Thus, in the present configuration, MHz-XPCS is sensitive to the motion inside the first coordination shell of the protein molecules in the dense solution.

Furthermore, it is interesting to examine the dynamics at dose values below the static damage threshold of 10 kGy obtained from the MHz-SAXS analysis. The diffusion constants are almost independent of the initial dose for a given dose rate. However, *D*_0_ displays a pronounced rate dependence and increases by almost a factor of four between the lowest and the highest dose rate (Fig. 4b). Illuminating a sample with highly intense X-ray pulses can lead to a temperature increase. Based on the X-ray beam size of 10 μm, we estimate that the generated heat dissipates with a time constant of 310 μs (see “Methods” section below), which is much longer than the measurement window covered by a *g*_2_-function here (20 μs). Thus, the illuminated sample volume does not cool down noticeably during a measurement and the maximum accumulated heat only depends on the fluence per pulse and the number of pulses illuminating the same sample volume which is equivalent to the accumulated dose.

**Fig. 5:**
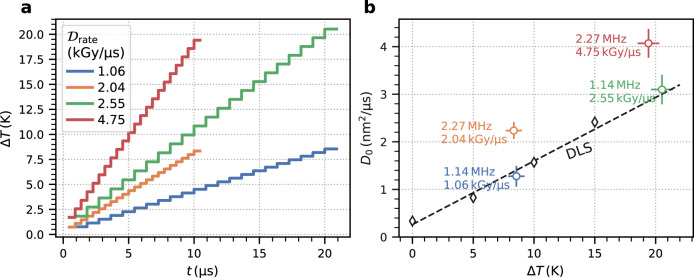
Effect of temperature on the dynamics. **a** Calculated temperature increase Δ*T* after 20 XFEL pulses for the four different dose rates. **b** Diamonds: Temperature dependence of diffusion coefficients measured by dynamic light scattering (DLS). The dashed black line is a linear fit to the data. Circles: diffusion constants determined via XPCS and minimum initial dose using the pulse frequency and dose rate indicated. The temperature assigned to the XPCS diffusion constants is estimated based on the respective temperature rise shown in **a**. The base temperature was *T*_0_ = 298 K for all XPCS measurements. Source data are provided as a Source Data file.

The increase of temperature for the different XFEL parameters is estimated following the model used by Lehmkühler et al.^1^ using a weighted average heat capacity of the constituents of *c*_*p*_ = 3.42 J g^−1^ K^−1^. With a maximum number of 3.82 × 10^8^ photons per X-ray pulse (see Table 1) the energy density is 5.5 mJ mm^−2^, which is an order of magnitude smaller than in the work of Lehmkühler et al.^1^. Fig. 5a displays the corresponding temperature rise during a pulse train with a temperature increase of Δ*T* ≈ 8 K after the low intensity pulse trains (blue and orange) and Δ*T* ≈ 19–21 K after the high intensity pulse trains (red and green). For comparison, we measured the temperature dependence of the equilibrium diffusion constant with dynamic light scattering (DLS), where the sample was equilibrated at each temperature before a measurement, and display the results in Fig. 5b. The values of *D*_0_ measured with MHz-XPCS and X-ray pulse repetition rates of 1.13 MHz are close to their equilibrium values obtained from DLS, when taking the XFEL-induced temperature increase into account. In contrast, employing higher XFEL frequencies of 2.26 MHz yields consistently higher diffusion coefficients which cannot be explained by a temperature increase alone. We hypothesize that the intense MHz XFEL pulses create a non-equilibrium state triggering processes on the sub-microsecond time scale. One example of such processes is the spatial homogenization of the aforementioned radiolysis products. The typical rates of secondary products such as OH^−^ radicals are on the order of microseconds^66,67^. Thus, on sub-microsecond time scales, the XFEL pulses simultaneously produce and probe a spatially inhomogeneous local distribution of the radiolysis products. The resulting chemical gradients, molecular repulsion due to dose rate-dependent protein charging, and possibly changes of the ionic strength of the solution, as well as damage to the PEG molecules, could contribute to the observed enhanced diffusive motion. Clearly, more systematic data and additional work by theory and simulation is needed to understand this XFEL-driven motion.

**Fig. 6:**
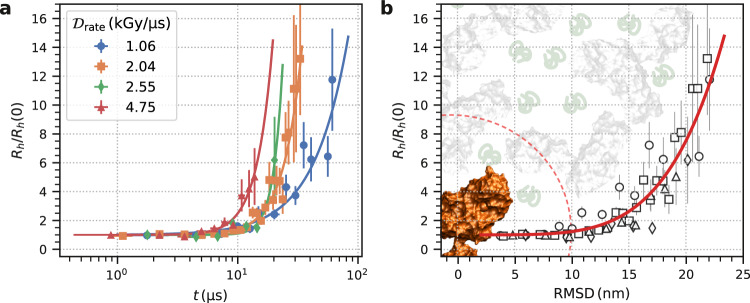
X-ray induced aggregation pathways. **a** Apparent hydrodynamic radii normalized to the initial value *R*_*h*_(0) as a function of measurement time for different dose rates. The solid lines are guides to the eye. **b** Apparent hydrodynamic radii normalized to the initial value *R*_*h*_(0) as a function of root mean square displacement (RMSD). The red solid line is a guide to the eye. The dashed red circle describes a sphere with a radius of about 10 nm and marks the space an Ig molecule can explore before the onset of aggregation. Source data are provided as a Source Data file.

The question arises why the faster dynamics at higher dose rates do not lead to a dose rate-dependent aggregation visible in the SAXS signal. We address this question by employing the Stokes–Einstein relation and estimating the temporal evolution of the relative changes to the apparent hydrodynamic radii via *R*_*h*_(*t*)/*R*_*h*_(0) = *D*_0_(0)/*D*_0_(*t*), where *D*_0_(0) and *R*_*h*_(0) represent the respective values at the minimum dose in Fig. 4. The increase of this ratio serves as an indicator for protein aggregation. Figure 6 shows that aggregation sets in earlier and develops faster for higher dose rates. For a dose rate of 1.06kGy μs^−1^, *R*_*h*_(*t*)/*R*_*h*_(0) has approximately doubled after (18.0 ± 0.9) μs and after (6.8 ± 0.6) μs for 4.75 kGy μs^−1^ (see Supplementary Fig. 4). Using the measured diffusion coefficients *D*_0_, we further calculate the time-dependent root mean square displacement (RMSD) $\sqrt{\langle {{\Delta }}{x}^{2}(t)\rangle }=\sqrt{6{D}_{0}t}$ of the proteins and plot *R*_*h*_(*t*)/*R*_*h*_(0) as a function of RMSD (see Fig. 6b). The data for the different dose rates collapse onto a single master curve (red line) indicating that the onset of aggregation mainly depends on the RMSD of the protein molecules. Higher dose rates induce faster movement of the proteins, and thus the RMSD necessary for aggregation is reached earlier. Figure 6b also reveals that aggregation sets in after a RMSD of about 10 nm and the space a single protein can explore in the crowded solution before that happens is indicated by a red dashed circle. This area is quite large considering that the sample is a densely packed solution of 250 mg ml^−1^, where the mean free path *l* between two molecules is typically smaller than their radius. We estimate $l=1/(n\pi {(2{R}_{h})}^{2})=\,{{\mbox{2.6}}}\,\,\,{{\mbox{nm}}}$ from the number density *n* and the molecular radius *R*_*h*_ = 5.5 nm of an Ig molecule which in turn implies an average number of contacts between proteins on the order of *N* = 〈Δ*x*^2^(*t*)〉/ *l* ^2^ ≈ 14 before aggregation sets in.

Our analysis indicates that aggregation is not strictly translational diffusion limited, but multiple contacts are necessary to attach two protein molecules to each other and form aggregates. This may hint towards the importance of specific interaction sites driving the aggregation process^68^. In addition, we note that unfolding processes, which increase the protein propensity to aggregate do also occur on time scales of microseconds^69^. Thus, the observed initial period of constant *R*_*h*_ points towards a minimum incubation time on the order of 10 μs before the proteins locally unfold or a time needed for rotational motion of molecules in order to allow activated sites to form local bonds. This incubation time and the minimum RMSD of 10 nm define a window of opportunity where dynamics can be measured in a *correlation before aggregation* scheme. Analogously to *diffraction before destruction* on femtosecond time scales^70–73^, *correlation before aggregation* will allow to obtain experimental information about the structural sample dynamics on (sub-)microsecond time scales before X-ray induced changes will become apparent in the XPCS signal. Additional data with more dose rates could allow developing novel methods to estimate the diffusion coefficients at zero dose rate^74^. However, the dose rate dependence might be highly dependent on the sample. On the other hand, developing the *correlation before aggregation* approach further would provide a window of opportunity, where with moderate doses and dose rates, the SNR is increased and the overall measurement time and sample consumption could be reduced.

Reducing the sample consumption is crucial for measuring particularly precious solutions or systems that exhibit phase transitions on microsecond time scales, e.g., biomolecular condensates, which is hard to repeat thousands of times^52,58,75^. Improving the experiments could be achieved for instance by making use of the self-seeding schemes which provide a much larger longitudinal coherence length. A larger longitudinal coherence length would allow for a larger beam size with similar measured speckle contrast yielding a lower photon density on the sample. This reduces the radiation damage to the sample and the amount of sample needed. It also increases the scattering volume and scattering intensity and thus strongly increases the SNR ratio^76^. Further technical improvements such as MHz detectors with smaller pixel size are needed to improve the SNR even further which allows extending the accessible *q*-range and lowering the dose rate needed.

Summarizing, we demonstrated that MHz-XPCS bears the potential to become a useful tool for measuring dynamics of biological macromolecules in solution on molecular length scales and on the time scales relevant for diffusive motion in cells. Importantly, our results indicate that taking the temperature rise of the solution into account allows for studying equilibrium dynamics within the first coordination shell of the molecules. Higher XFEL frequencies drive the dynamics and lead to increasing diffusion coefficients and aggregation which sets in after a time window of 10 μs. We refer to this approach as correlation before aggregation which allows to capture protein dynamics in solution before the manifestation of X-ray-induced effects. Additional experiments and simulations are needed to fully understand the underlying physics of the involved processes. Understanding the observed dose rate dependence of the diffusion process involves accurate knowledge of a number of yet unknown factors, such as the role of the interaction potentials, concentration, solvent chemical composition, and size and masses of the proteins. Resolving these properties and the role of radiolysis processes and their products in this context will determine the best data acquisition strategies for measuring the unperturbed dynamic properties.

## Methods

### Sample preparation

The sample preparation followed a procedure provided by the literature^51^. Polyclonal bovine immunoglobulin (purity ≥99%, Sigma-Aldrich, SRE0011), PEG 1000 (Sigma-Aldrich, 81188), NaCl (Merck 106404), HEPES (Roth, HN78), and NaN_3_ (Sigma-Aldrich, S8032) were used as received. All solutions were prepared in a buffer of composition 20 mM HEPES pH = 7.0, 2 mM NaN_3_, using degassed Milli-Q water (Merck Millipore 18.2 MΩ cm). The concentration of the immunoglobulin stock solution was assessed by UV absorption at 280nm, using an extinction coefficient of *e*_280_ = 1.4 mL mg^−1^ cm^−1^ with a Cary 50 UV–Vis spectrophotometer. The experimental phase diagram of this system has been established in our previous work^51^. The "parent solution” was equilibrated for about 24 h at 294 K and then briefly centrifuged, resulting in a clear dense and a dilute phase, separated by a sharp meniscus. The parent solution composition was immunoglobulin 200 mg/mL, PEG 12% w/v and NaCl 150 mM. The dense liquid phase was used for XPCS measurements with a concentration of roughly 250 mg mL^−1^.

### Measurement protocol

The sample was moved continuously through the X-ray beam to refresh the sample volume between trains. The translation motor speed was 0.4 mm s^−1^ leading to an absolute sample translation of 40 μm between successive trains (a train arrives every 100 ms). X-ray generated heat diffuses with a thermal diffusion constant of *D*_*T*_ = 0.143 mm^2^ s^−1^. Assuming homogeneous heat diffusion in radial direction perpendicular from the beam, after *τ*_train_= 100 ms the heat diffused in a cylinder with radius $\sqrt{6{D}_{T}{\tau }_{{{{{{{{\rm{train}}}}}}}}}}\; \approx$ 293 μm. The illuminated volume of the next train is then 10 μm^2^/293 μm^2^ ≈ 0.1% of that volume. Therefore, beam-induced heating effects generated by the previous train can be neglected for the following illumination.

### Calculation of the absorbed dose and dose rate

On the time scale of an individual X-ray pulse (≤50 fs^50^) and with maximum flux, the peak dose rate can reach several hundred MGy per pulse^77^. Utilizing MHz repetition rates and high attenuation, the X-ray pulses are delivered on (sub-) microsecond time scales such that an average dose rate on the order of several kGy per microsecond can be reached. Correspondingly, synchrotron sources produce typical average dose rates of kGy per second.

In order to quantify the amount of energy absorbed by a certain sample mass, we calculate the dose, ${{{{{{{\mathcal{D}}}}}}}}({N}_{p})$, absorbed by the sample after *N*_*p*_ pulses:

5 $${{{{{{{\mathcal{D}}}}}}}}({N}_{p})={\left\langle \mathop{\sum }\limits_{i=1}^{{N}_{p}}\frac{{{{\Phi }}}_{c}^{j}(i){E}_{c}A}{{z}^{2}{d}_{s}\rho }\right\rangle }_{{{{{{{{\rm{j}}}}}}}}},$$

where $A=(1-{{{{{\rm{e}}}}}}^{-{d}_{s}/{\mu }_{{{{{{{{\rm{eff}}}}}}}}}})$ denotes the sample absorption where *d*_*s*_ = 1.5 mm is the sample thickness and *μ*_eff_ = 1.35 mm is the effective attenuation length of the solution calculated as the weighted harmonic mean of the individual components^52,78^, *E*_*c*_ = 9 keV the photon energy, ${{{\Phi }}}_{c}^{j}(i)$ the number of photons in pulse *i* in train *j*, *ρ* = 1.09 g cm^−3^ is the sample density, and *z* = 10 μm is the beam size. 〈… 〉_j_ denotes an average over trains. The dose is measured in Gray (1 Gy = 1 J kg^−1^).

To account for SASE related intensity fluctuations, the average dose rate is calculated as the average dose absorbed by the sample after ten pulses divided by the delay between two successive pulses, *τ*_*p*_:

6 $${{{{{{{{\mathcal{D}}}}}}}}}_{{{{{{{{\rm{rate}}}}}}}}}=\frac{{{{{{{{\mathcal{D}}}}}}}}({N}_{p}=10)}{10{\tau }_{p}}.$$

### Error bar calculation

The error bars of the correlation functions in Fig. 3 are calculated as the standard deviation of the fluctuations of the contrast values within a *q*-bin. This standard deviation is used to calculate the weighted average over trains and times in Eqs. (2) and (3). These error bars are used in the fits for the estimation of the parameters. The error bars in the following plots, which display the fit results of the correlation functions, indicate the parameter uncertainty obtained from the fits using least-squares minimization.

### X-ray induced heating

The relaxation time of heat diffusion used in Fig. 5 to estimate the X-ray induced heating is calculated as *τ*_heat_ = *c*_*p*_*ρz*^2^/(2*k*_*w*_) = 310 μs, where *c*_*p*_ = 3.42 J g^−1^ K^−1^ is the heat capacity of the solution and *k*_*w*_ = 0.6 W m^−1^ K^−1^ is the thermal conductivity. The heat capacity is calculated by an average of the heat capacities of water, PEG, and IgG weighted with their volume fractions. The temperature increase induced by a pulse with energy *E*_*p*_ is ${{\Delta }}{T}_{\max }=4\log (2){E}_{p}/(2\pi {c}_{p}\rho {z}^{2}{\mu }_{{{{{{{{\rm{eff}}}}}}}}})$. This formalism leads to values from Δ*T* = 0–21 K depending on *E*_*p*_ and the number of X-ray pulses (see Fig. 5). It should be noted that 1% and less of the maximum possible *E*_*p*_ at MID has been used to reduce beam-induced effects.

### Reporting summary

Further information on research design is available in the Nature Research Reporting Summary linked to this article.

## Supplementary information

Supplementary Information

Reporting Summary

## Data Availability

The data are available from the authors upon request. Source data are provided with this paper.

## Source data

Source Data

## Code availability

Analysis scripts are available from the authors upon request.
