# Supplementary material for: Resolving molecular diffusion and aggregation of antibody proteins with megahertz X-ray free-electron laser pulses
Source: Nat Commun. 2022 Sep 21;13:5528. doi: 10.1038/s41467-022-33154-7 (PMC9490738; doi:10.1038/s41467-022-33154-7)
Supplement: Supplementary file 1 — Supplementary Information [file 41467_2022_33154_MOESM1_ESM.pdf]

Supplementary Information: Resolving molecular diffusion and aggregation of antibody proteins with megahertz X-ray free-electron laser pulses

Mario Reiser et al.

Supplementary Discussion

Coherence and Speckle Contrast

The experimental speckle contrast, β_{exp} depends on nearly all experimental parameters such as pixel size speckle size, beam size, sample thickness, momentum transfer q, the transverse and longitudinal coherence properties of the X-rays, etc. β_{exp} can be calculated as the product of the longitudinal contrast factor, β_l, and the transverse contrast factor, β_t:

$$\beta_{\mathrm{exp}} = \beta_l \beta_t \,. \tag{1}$$

For XFELs, the model described in Hruszkewycz *et al.* [1] is often employed to estimate $\beta_l(q)$. A detailed description of the mathematical formalism can be found in the supplementary material of [1]. Lehmkühler *et al.* [2] show that the speckle contrast at EuXFEL can be described by this model as well. β_l is determined by the energy bandwidth, $\Delta E/E$, which can be decreased by using a seeded beam or a monochromator. Both were not available for this experiment. Instead, the pink SASE beam was used with an energy bandwidth of $\Delta E/E \approx 2 \times 10^{-3}$. For the transverse coherence factor, $\beta_t \approx 0.5$ was found for different XFELs including European XFEL [2–5]. Eventually, the following model was used to describe the data

$$\beta_{\mathrm{exp}}(q) = 0.5\, \beta_l(q) \,. \tag{2}$$

Fig. 1 displays $\beta_{\mathrm{exp}}(q)$ as calculated by Eq. (2) as function of the momentum transfer assuming

*mario.reiser@fysik.su.se
†f.perakis@fysik.su.se
‡christian.gutt@uni-siegen.de

a beam size of $10\,\mu\text{m}$, a sample-detector distance of $7.46\,\text{m}$, a photon energy of $9\,\text{keV}$, a sample thickness of $1.5\,\text{mm}$, a pixel size of $200\,\mu\text{m}$, and a bandwidth of 2×10^{-3} and 1×10^{-4}, respectively. The blue line indicates the contrast during the experiment where the black dots are the corresponding values used to model the correlation functions. The orange line shows the increased speckle contrast when using a smaller bandwidth.

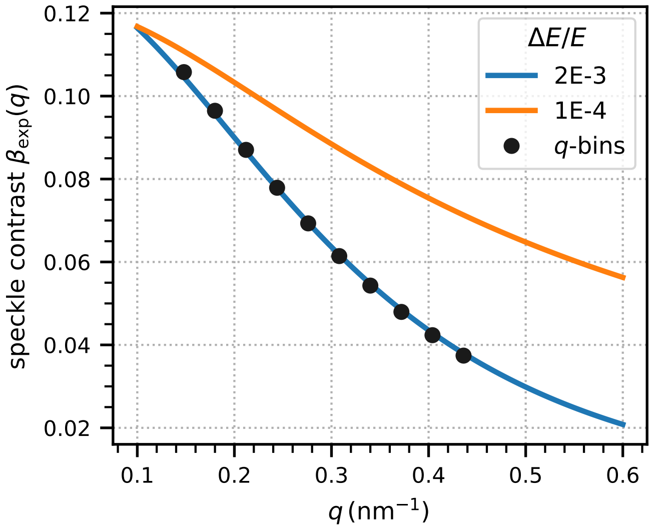

Supplementary Fig. 1: **Speckle contrast for different energy bandwidths**. $\Delta E/E = 2 \times 10^{-3}$ corresponds to the SASE bandwidth and $\Delta E/E = 1 \times 10^{-4}$ to a monochromatic beam. The black points indicate the contrast values in the q-bins used to fit the experimental data.

Signal-to-Noise Ratio

Following the work of Falus *et al.* [6], the XPCS signal-to-noise ratio, R_{sn}, can be calculated as

$$R_{sn} = \beta I \sqrt{N_p N_{\text{trains}} N_{\text{pix}}}\,, \tag{3}$$

where β is the speckle contrast, I is the intensity in numbers of photons per pixel, $N_p = 20$ is the number of pulses or images used to calculate the first point of the correlation functions, N_{trains} is the number of trains that are averaged, $N_{\text{pix}} = 7494$ is the number of pixels in the q-bin where the correlation function is calculated.

Fig. 2 displays R_{sn} of the first point of the correlation functions at $0.148\,\mathrm{nm}^{-1}$ as a function of the total number of X-ray pulses, where each of the N_{trains} illuminated a fresh sample volume. The data have been rebinned along the abscissa and the error bars indicate the standard deviation within each bin. In addition, the total measurement time is indicated assuming that every pulse is used for the analysis. The measurement time obviously depends on both machine performance and filtering criteria applied during the experiment as not every train might be usable for the analysis, e.g., because of very low intensity. In Fig. 2, it is assumed that every train is used for the analysis. In our experiment about $20\,\%$ of the trains were discarded. The visible fluctuations in R_{sn} are probably related to fluctuating instrument parameters and varying machine performance.

The primary advantage of using a monochromatic beam is that it would allow for a larger beam size with similar contrast, hence yielding a lower photon density on the sample. This reduces the radiation damage to the sample and the amount of sample needed. It also increases the scattering volume and scattering intensity and thus strongly increases the signal-to-noise ratio [7].

Azimuthal Dependence of the Scattering Signal

Fig. 3 displays the scattering signal from Fig. 2 in the main manuscript at $q = 0.4\,\mathrm{nm}^{-1}$ as a function of the azimuthal angle ϕ for different doses. The data have been normalized to the average value of each curve to exclude any effect on the average scattering signal. The small kinks can be attributed to the AGIPD detector. The signal does not exhibit any sign of anisotropy within the measurement accuracy. Furthermore, the shape of the curves does not change as a function of absorbed dose indicating that the signal stays isotropic throughout the measurements.

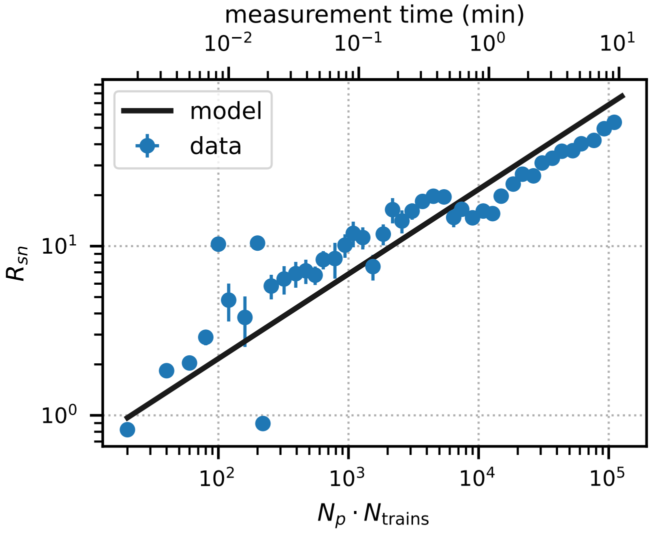

Supplementary Fig. 2: **Signal-to-noise ratio as a function of the number of X-ray pulses.** The signal-to-noise ratio of the experimental data compared with the estimations of Eq. (3). On the top, the total measurement time is indicated assuming that every train measured is suitable for the analysis.

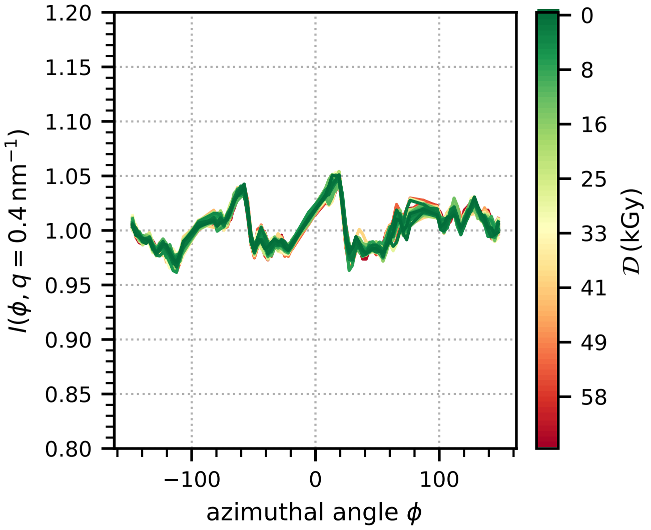

Supplementary Fig. 3: **Azimuthal scattering intensity.** Integrated scattering signal at $q = 0.4\,\mathrm{nm}^{-1}$ as a function of azimuthal angle ϕ. $\phi = 0$ corresponds to the horizontal direction. The data have been normalized to the average value of each curve. The color indicates the absorbed dose. The lines of different doses are overlapping almost perfectly.

Dose dependence of the Hydrodynamic Radius

We plot the normalized hydrodynamic radius R_h as a function of dose in Fig. 4a and as a function of root mean squared displacement in Fig. 4b for comparison as in the main manuscript. From fitting the data of the lowest and highest dose rate (dashed lines) with an exponential model we conclude that R_h increases by a factor of two after (19.2 ± 1.0) kGy for a dose rate of 1.06 kGy μs^{-1} and after (32 ± 3) kGy for a dose rate of 4.75 kGy μs^{-1}. These values correspond to starting times of (18.1 ± 0.9) μs and (6.8 ± 0.6) μs, respectively.

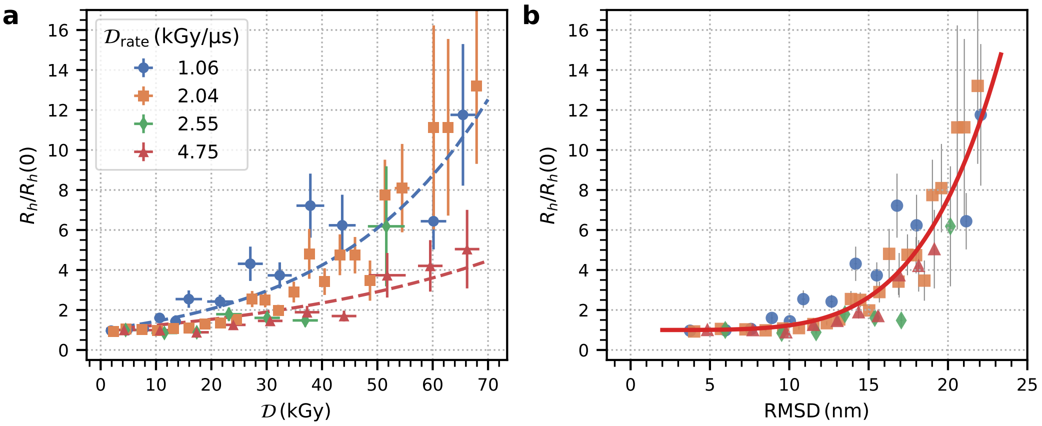

Supplementary Fig. 4: **Dose dependence of the hydrodynamic radius.** (**a**) Normalized hydrodynamic radius R_h as function of dose. (**b**) Normalized hydrodynamic radius R_h as function of root mean squared displacement. The dashed lines in (**a**) indicate fits with an exponential model. The error bars describe the fit accuracy.

Supplementary References

1. Hruszkewycz, S. O. *et al.* High Contrast X-ray Speckle from Atomic-Scale Order in Liquids and Glasses. *Phys. Rev. Lett.* **109,** 185502 (2012).

2. Lehmkühler, F. *et al.* Emergence of Anomalous Dynamics in Soft Matter Probed at the European XFEL. *Proc. Natl. Acad. Sci. U.S.A.* **117,** 24110–24116 (2020).

3. Madsen, A. *et al.* Materials Imaging and Dynamics (MID) Instrument at the European X-ray Free-Electron Laser Facility. *J. Synchrotron Radiat.* **28,** 637–649 (2021).

4. Lehmkühler, F. *et al.* Single Shot Coherence Properties of the Free-Electron Laser SACLA in the Hard X-ray Regime. *Sci Rep* **4,** 5234 (2014).

5. Alonso-Mori, R. *et al.* The X-ray Correlation Spectroscopy Instrument at the Linac Coherent Light Source. *J Synchrotron Rad* **22,** 508–513 (2015).

6. Falus, P., Lurio, L. B. & Mochrie, S. G. J. Optimizing the Signal-to-Noise Ratio for X-ray Photon Correlation Spectroscopy. *J. Synchrotron Radiat.* **13,** 253–259 (2006).

7. Möller, J., Sprung, M., Madsen, A. & Gutt, C. X-Ray Photon Correlation Spectroscopy of Protein Dynamics at Nearly Diffraction-Limited Storage Rings. *IUCrJ* **6,** 794–803 (2019).
